# Efficacy of Local Minocycline Agents in Treating Peri-Implantitis: An Experimental In Vivo Study in Beagle Dogs

**DOI:** 10.3390/pharmaceutics12111016

**Published:** 2020-10-23

**Authors:** Sung-Wook Yoon, Myong-Ji Kim, Kyeong-Won Paeng, Kyeong Ae Yu, Chong-Kil Lee, Young Woo Song, Jae-Kook Cha, Ui-Won Jung

**Affiliations:** 1 Department of Periodontology, Research Institute for Periodontal Regeneration, Yonsei University College of Dentistry, Seoul 03772, Korea; sungwook08@yuhs.ac (S.-W.Y.); mjikim212@yuhs.ac (M.-J.K.); kwp9310@yuhs.ac (K.-W.P.); tigger09@yuhs.ac (Y.W.S.); chajaekook@yuhs.ac (J.-K.C.); 2 College of Pharmacy, Chungbuk National University, Cheongju 28165, Korea; serom001@naver.com (K.A.Y.); cklee@chungbuk.ac.kr (C.-K.L.)

**Keywords:** chitosan-alginate microspheres, drug sustainability, locally applied antibiotics, peri-implantitis, pharmacodynamic

## Abstract

Background: Local delivery agents (LDA) have the advantage of delivering the antibiotics at high concentrations to the targeted sites. However, the constant flow of gingival crevicular fluids and saliva may restrict their efficacy. Therefore, the drug sustainability and pharmacodynamic properties of any proposed LDA should be evaluated. Methods: Four dental implants were placed unilaterally in the edentulous mandible of six beagle dogs. Peri-implantitis were experimentally induced using silk-ligatures. Each implant was randomly allocated to receive one of the following four treatments: (i) MC (Chitosan-alginate (CA) minocycline), (ii) MP (CA-without minocycline), (iii) PG (Polyacrylate-glycerin minocycline), and (iv) Control (mechanical debridement only). Mechanical therapies and LDAs were administered into the gingival sulcus two times at a 4-week interval. Drug sustainability as well as clinical, radiographical, and immunohistochemical (IHC) analyses were conducted to evaluate the efficacies of treatments. Results: Reduced mean probing depth was observed in all of the test groups after the second delivery. A minimal marginal bone level change was observed during the treatment period (MP (−0.06 ± 0.53 mm) to PG (−0.25 ± 0.42 mm)). The distribution of IHC cell marker analysis of all targeted antibodies ranged from 6.34% to 11.33%. All treatment outcomes between the test groups were comparable. A prolonged retention of LDA was observed from CA microspheres (MC and MP) at both administrations (*p* < 0.017) and prolonged sustainability of bacteriostatic effect was observed from MC compared to PG after the second administration (*p* < 0.05). Conclusions: Prolonged retention of CA microspheres was observed and the longer bacteriostatic effect was observed from the MC group. Mechanical debridement with adjunct LDA therapy may impede peri-implantitis progression, however, prolonged drug action did not lead to improved treatment outcome.

## 1. Introduction

Peri-implantitis is an inflammatory peri-implant disease with the evidence of bleeding on probing, increased pocket probing depth, and progressive supporting bone loss [1]. Peri-implantitis is present in 20% of the population with dental implants and the prevalence rate is found to increase as the implants are retained for a longer period [2]. Therefore, an effective modality to manage peri-implantitis is necessary owing to the fact that the number of implants is ever-increasing.

Peri-implantitis may be triggered by various surgical and prosthetic risk factors including implant malposition, failed bone reconstruction, cement remnants, and presence of overloading [3,4]. Among several possible causes of peri-implantitis, bacterial infection in dental plaques is the major predisposing factor for the initiation and progression of peri-implantitis [5]. Various modes of mechanical and chemical therapies have been suggested to treat peri-implantitis [6,7]. In areas where deep pockets are created, surgical approaches are suggested for the instrumental access to thoroughly remove inflammatory tissues and pathogenic contaminants [8]. Nowadays, various instruments such as different elements (e.g., plastic and titanium) of curettes and ultrasonic tips, air-powder abrasive, and laser devices are available to improve the efficiency of implant mechanical therapy [9,10,11]. Although ultrasonic tips are highly effective, titanium brushes with various shapes and air-powder abrasive devices have been advocated for their accessibility and less topographical changes on the implant surface [9,10]. Moreover, a recent review article had reported that antimicrobial photodynamic therapy has the ability to decrease the count of peri-implant pathogens [11].

However, mechanical debridement alone has limitations in approaching the deepest sites due to anatomical characteristics of the implants and defects [12]. In cases where mechanical therapy is not effective, adjunctive chemical therapies can be selected [13]. Chemical therapies are broadly used from primary to adjunctive therapies in the treatment of bacterial-induced peri-implant disease. Therefore, antibiotic treatment means to remove bacterial contaminant in the dental plaque adjunct to mechanical debridement and ultimately aims to convert the pathological bacterial distribution in the periodontal pocket into a normal bacterial distribution [14].

Local delivery of agent (LDA) has the advantage of effectively delivering the antimicrobial agent into the gingival sulcus promptly with higher concentration over systemic delivery in a peri-implant bone defect [15]. Various antibiotics including tetracycline, minocycline, chlorohexidine, and metronidazole have been evaluated for local delivery in forms of soluble, non-degradable or biodegradable polymers. Soluble carriers fail to penetrate periodontal pockets deeper than 3 mm and nondegradable vehicles require additional visits for the removal [16]. Therefore, biodegradable polymers with an adequate amount of sustainability are selected as an applicable candidate for the adjunctive therapy. A recent randomized clinical study showed improved clinical outcomes and supporting bone levels from peri-implantitis defects that repeatedly used adjunctive minocycline-loaded LDA combined with surgical treatment when compared to the placebo ointment applied group [17].

Despite the preference of biodegradable LDA in treating peri-implant bone defects, their application is still beset by insufficient maintenance of drug concentration. Polyacrylate-glycerin (PG) microspheres have been advocated for their rapid release of the antibiotic agents in the microsphere and instinct effects [18]. Other types of carriers include a polysaccharide microsphere composed of chitosan-alginate (CA) that can perform a controlled release of antibiotics [19]. The authors speculate that a slowly degraded microsphere could sustain optimal therapeutic concentration for a longer period and could ultimately improve the treatment outcome. Therefore, the null hypotheses were as follows: 1. CA and PG will exhibit equivalent carrier and bacteriostatic effect sustainability; 2. All treatment groups will present comparable treatment outcomes.

A recent preclinical study has investigated the drug sustainability of PG and CA microspheres and their treatment outcomes in an experimentally induced peri-implant mucositis (mucositis) model in dogs [20]. This serial study aims to evaluate the treatment efficacies of the two different LDA carriers in respect of differential drug sustainability in a more advanced inflammatory environment—an experimentally induced peri-implantitis.

## 2. Materials and Methods

### 2.1. Experimental Animals

This experimental in vivo study was approved by the International Animal Care and Use Committee, Yonsei Medical Research Centre, Seoul, Korea (Permission No. 2017-31-0547, 17 October 2017). Six male beagle dogs (weigh 15 kg) about one-year-old were used. The dogs were maintained under the standard lab condition with the supervision of professional veterinarians at Avison Biomedical Research Centre of Yonsei University, Seoul, Korea. The study was designed following the ARRIVE guidelines [21].

### 2.2. Sample Size Calculation

The sample size of this study was calculated based on the previous study [22] that evaluated the mean duration of drug action (>1 µg/mL) of minocycline-loaded nano-particles (12 days) and minocycline-loaded poly(meth)acrylate-glycerine microsphere (8 days) with significance level of 10% and 90% statistical power (Standard deviation = 1.5). A minimal sample size of four in each group was calculated using a statistical power analyses program (G*Power, Heinrich Heine University, Düsseldorf, Germany). Allowing for any possible dropouts, the sample size for each group was determined to be six.

### 2.3. Experimental Procedures

#### 2.3.1. Extraction, Implant Placement, and Peri-Implantitis Induction

**Figure 1:**
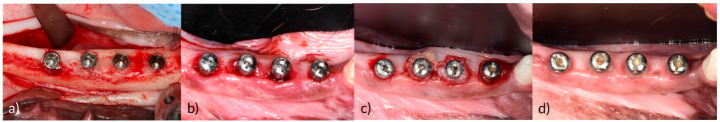
Clinical photographs taken at (**a**) the surgery, (**b**) after ligature removal, (**c**) at the first LDA administration, and (**d**) at 4 weeks after the second administration.

**Figure 2:**
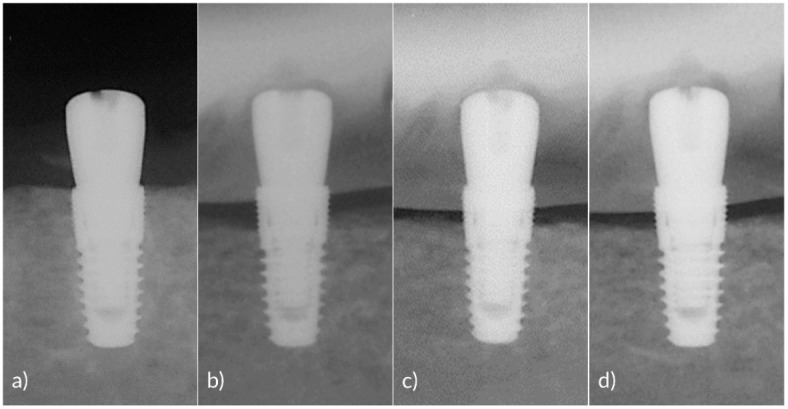
Peri-apical radiographs taken at (**a**) implantation (Surgery), (**b**) after inducing peri-implantitis (Baseline, T1), (**c**) 4 weeks after the first administration (T2), and (**d**) 4 weeks after the second administration (T3).

Second premolars to first molars on the unilateral side of the mandible were extracted from each dog. After two months of the healing period, four internal type implants (3.6 × 8.0 mm Implantium, Dentium, Suwon, Korea) were installed (Surgery) on the extracted sites at eqi-crest level (Figure 1a). The healing abutments (4.0 × 2.0 mm, Dentium) were then connected to the implants. After 4 weeks of osseointegration period, silk ligatures (2-0 Gingi-pak^®^, Camarillo, CA, USA) were placed into the gingival sulcus around healing abutments for 6 weeks to induce peri-implantitis [23]. During the peri-implantitis induction period, oral hygiene control was discontinued, and plaque accumulation was induced with a soft diet. To assess crestal bone level changes, peri-apical radiographs were taken with a portable X-ray (DIOX-602, Digimed, Seoul, Korea) immediately after implantation, 1, 2, and 6-weeks post-installation. After peri-implantitis was induced, inserted silk ligatures were removed from the gingival sulcus (Figure 1b) and 2 weeks of recovery period was given.

#### 2.3.2. Localized Drug Agent Delivery

The four treatment groups were as follows:

1. 0.5 g of minocycline hydrochloride with CA microsphere (MC; Minocline^®^, Dongkook Pharmaceutical, Seoul, Korea);
2. Placebo CA microsphere ointment without minocycline hydrochloride (MP; Minocline Placebo, manufactured by Dongkook pharmaceuticals);
3. 0.5 g of minocycline hydrochloride with PG microsphere—obtained from Sunstar (PG; Periocline^®^, Sunstar, Osaka, Japan);
4. Mechanical debridement only (Control).

Firstly, the sequence of the treatment allocated to each implant in a dog was generated using a computer random number generator. The sequences were then rotated in a block manner for each dog. A professional mechanical debridement with an ultrasonic scaler (Osung MND, Seoul, Korea) was conducted to every implant before LDA administration. The LDAs were repeatedly administered two times at 4 weeks interval by means of a polyethylene syringe (Figure 1c).

### 2.4. Outcome Variables

- Primary outcome: clinical, radiographical, and immuhohistochemical outcomes.
- Secondary outcome: LDA carrier sustainability, bacteriostatic effect durability.

### 2.5. Clinical Outcomes

Clinical parameters of pocket probing depth (PPD), bleeding on probing (BOP), gingival index (GI) [24], and plaque index (PLI) [25] were measured at the baseline (T1), 4 weeks after the first administration (T2), and 4 weeks after the second administration (T3) by a single examiner (M.J.K.) to evaluate the interval changes and severity of the inflammation.

### 2.6. Radiographical Outcomes

A set of peri-apical radiographs were taken at the surgery, T1, T2, and T3 to measure the marginal bone level changes over the treatment period (Figure 2a–d). All of the implants were placed on the alveolar bone crest level, therefore abutment-fixture junction (A/F) was taken as a reference line. The mean distance from A/F to marginal bone crest at mesial and distal sides of all implants were recorded using Image J (National Institutes of Health, Bethesda, MD, USA) [26].

### 2.7. Histological Preparation and Immunohistochemical (IHC) Analysis

Histological preparation and IHC cell-marker analysis followed the same protocol as described in the previous experimentally induced mucositis study [20]. In brief, tissue blocks of all implants were prepared and buccal and lingual aspects parallel to the implants were collected using the fracture technique [27]. The sectioned blocks were then processed with IHC staining [28]. Three panels of primary antibodies were used in IHC staining—CD3 (Ab828, Abcam, Cambridge, UK), CD20 (PA5-16701, Thermo Fisher Scientific, Seoul, Korea), and IgG (PAA544Ca01, Could-Clone Corp., Katy, TX, USA) which are specific to T cells, B cells, and Plasma cells, respectively [26] (Appendix A Table A1).

**Figure 3:**
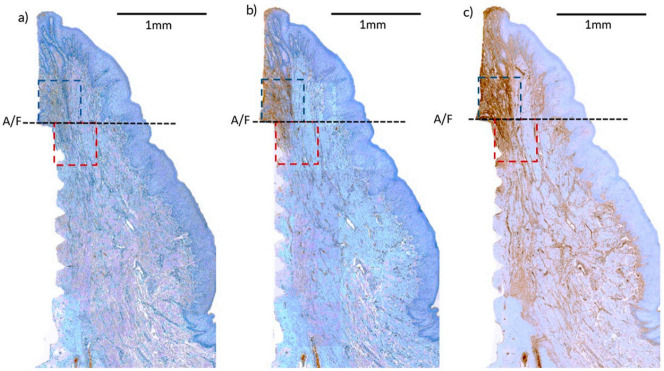
IHC staining of an implant using fracture technique (20× magnification). (**a**) CD3; (**b**) CD20; (**c**) IgG. Upper (Blue) and lower (Maroon) region of interest are labelled. A/F—abutment-fixture junction.

The digital images of the specimens were obtained (Panoramic 250 Flash III, 3D, Case Viewer 2.0, 3D Histech, Budapest, Hungary) and the intensities of positive cells were detected using the IHC profiler, Image J (National Institutes of Health, Bethesda, MD, USA) [29]. Areas of 0.5 × 0.5 mm above and below the A/F were analyzed (Figure 3).

### 2.8. Drug Sustainability Evaluation

#### 2.8.1. LDA Carrier Sustainability Evaluation

To evaluate the sustainability of LDA carrier, the healing abutments were retired from all implants from MC, MP, and PG groups and any presence of LDA residues at subgingival areas of healing abutments were circumferentially inspected in plain sight. Carrier sustainability was evaluated at days 14 and 28 after the first administration and days 1, 3, 7, 14, and 28 after the second administration.

#### 2.8.2. Bacteriostatic Effect Evaluation 

To evaluate the bacteriostatic effect longevity, GCF, and residual LDA samples (if present) were collected from MC, PG, and Control group implants. Gingival sulcus of mesial, distal, buccal, and lingual areas of the healing abutments was scraped with a single stroke using a Gracey curette (Osung MND, Seoul, Korea). The samples were collected on days 1, 3, 7, 14, and 28 after each LDA administration. The collected samples were promptly contained in centrifuge plastic tubes containing 200 µL of distilled water and stored at −80 °C in a freezer.

The broth dilution assay was carried out in a single-blinded manner following the standard protocol [30]. In brief, the collected samples were serially diluted in two-folds using phosphate-buffered saline (LPS solution, Daejeon, Korea) and each diluted solution (100 µL) and 100 µL of microbial rich broth (5 µL of *S. aureus* (2.5 × 10^7^ CFU) with 95 µL of LB broth (2x LB broth, BD Diagnostics, Sparks, MD, USA)) was subsequently added to a 96-well plate [20]. The solutions were incubated for 24–48 h at 37 °C, and the bacterial cell growth was evaluated at 600 nm using a microplate reader (SpectraMax M2, Molecular Devices, San Jose, CA, USA).

### 2.9. Statistical Analysis

A standard statistical software (SPSS version 25, IBM, NY, USA) was used in the analysis. The mean values of each group were calculated for the carrier sustainability and bacteriostatic longevity. The mean values of each implant were calculated in clinical, radiographical, and IHC cell-marker analysis. Due to the smallness of the sample, a non-parametric Kruskal–Wallis test was performed to compare the carrier and bacteriostatic effect sustainability after each administration and to compare IHC cell-marker intensity. If the results were significant (*p* < 0.05), Mann–Whitney U test was performed as a post-hoc test with the significance criterion adjusted according to Bonferroni’s method (*p* < 0.017). For the clinical and radiographical results, Kruskal–Wallis test (*p* < 0.05) was conducted to examine the differences between the groups at T1, T2, and T3, while Wilcoxon-signed-rank test (*p* < 0.05) was applied to assess treatment outcomes within each group at T1, T2, and T3.

## 3. Results

### 3.1. Number of Animals and Implants Analyzed

Results of all six dogs were included in the analysis. No systemic adverse events were observed in this study. Total of 24 implants (six implants per group) were included in the analysis.

### 3.2. Clinical Findings

Mean PPD, GI, BOP (%), and PLI recorded at Baseline (T1), T2, and T3 are listed in Table 1. Mean PPD was significantly reduced within all the groups at T3 compared to T1 and T2 (*p* = 0.027 for all groups). MC was the only group that showed a significant reduction of mean PPD between T1 and T2 (*p* < 0.05). Mean PLI was also significantly reduced within MC, MP, and PG groups at T3 compared to T1 and T2 (T1-T3: (MC: *p* = 0.028, MP and PG: *p* = 0.027); T2-T3: (MC and MP: *p* = 0.027, PG: *p* = 0.026)) while the Control group showed a significant reduction of PLI between T1 and T3 (*p* = 0.027). PG and Control group showed significantly reduced BOP (%) at T2 and T3 compared to T1 (*p < 0.05)* and PG was the only group that presented significantly improved GI at T2 and T3 compared to T1 (*p < 0.05)*.

### 3.3. Radiographic Analysis

**Figure 4:**
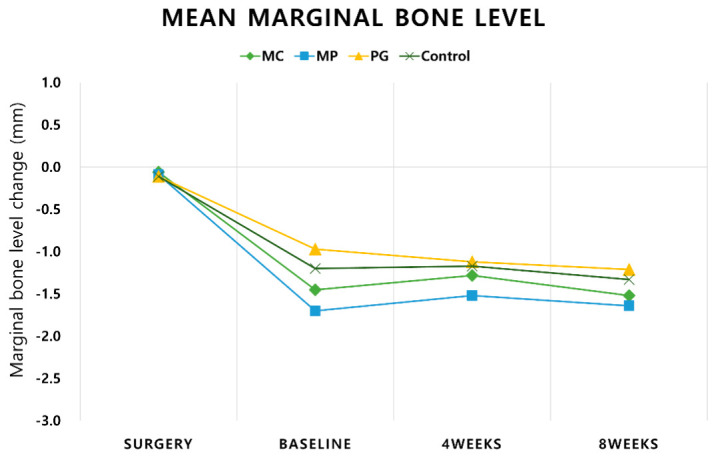
Mean marginal bone level change (mm).

Mean marginal bone loss of −0.9 mm (PG) to −1.6 mm (MP) occurred after inducing peri-implantitis with a ligature (Figure 4, Appendix A Table A2). Minimal mean marginal bone gain occurred from MC (0.17 ± 0.28 mm) and MP (0.12 ± 0.29 mm) while PG (−0.15 ± 0.24 mm) presented negligible mean marginal bone loss after the first delivery. Minimal mean marginal bone level change was also observed after the second delivery ranging from −0.06 ± 0.29 mm (MP) to −0.024 ± 0.25 mm (MC). All results were comparable between the groups.

### 3.4. Immunohistochemical (IHC) Analyses

Targeted cell marker analyses for CD3, CD20, and IgG are summarized in Table 2. CD3, a T-cell antibody, has a total mean positive score in the range of 6.34% (Control) to 9.29% (MP). CD20, a B-cell antibody, has a total mean positive score in the range of 7.85% (MP) to 8.55% (Control). IgG, a B-cell and Plasma cell antibody, has a total mean positive score in the range of 6.59% (MC) to 11.33% (Control). All groups showed a comparable positive score at upper and lower ROI. The positive scores of upper and lower ROI within the group were also comparable.

### 3.5. Drug Sustainability Evaluation

#### 3.5.1. Carrier Sustainability

**Figure 5:**
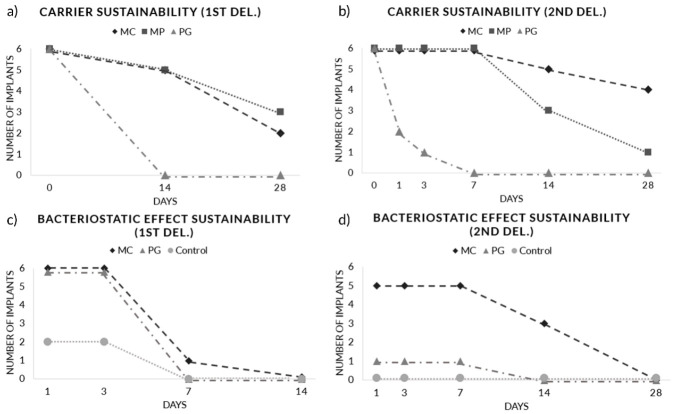
Carrier and bacteriostatic effect sustainability evaluation. (**a**) Carrier sustainability after the first administration, (**b**) carrier sustainability after the second administration, (**c**) bacteriostatic effect sustainability after the first administration, (**d**) bacteriostatic effect sustainability after the second administration.

Macroscopic evaluation of carrier sustainability showed significantly prolonged LDA retention of CA carrier (MC and MP) compared to PG microsphere at both administrations. During the first delivery, five implants (out of six) from MC (16.3 ± 10.5 days, *p* = 0.015, vs. PG) and MP (18.7 ± 11.4 days, *p* = 0.015, vs. PG) remained at day 14 while no LDA retention was observed from PG at day 14 (0.00 ± 0.00 days) (Figure 5a). After the second administration, five implants from MC (22.2 ± 9.30 days, *p* = 0.002, vs. PG) and three implants from MP (12.8 ± 8.18 d *p* = 0.002, vs. PG) groups remained at day 14 while PG (0.67 ± 1.21 days) showed significantly low LDA retention rate when compared to CA carriers (Figure 5b).

#### 3.5.2. Bacteriostatic Effect Sustainability

The longevity of LDA bacteriostatic effects was evaluated using the broth dilution assay. During the first delivery, all six implants from MC and PG showed bacteriostatic effect at day 3 and one implant from MC showed bacteriostatic effect up to day 7 (MC (3.67 ± 1.63 days), PG (3.0 ± 0.0 days); *p* = 0.70) (Figure 5c). During the second delivery, a prolonged bacteriostatic effect was observed in MC (9.33 ± 5.72 days) compared to PG (1.17 ± 2.86 days; *p* < 0.05) (Figure 5d). Five implants from MC showed a bacteriostatic effect on day 7 and that of three implants was effective until day 14. On the other hand, only one implant from PG showed a bacteriostatic effect until day 7.

## 4. Discussion

This study evaluated the sustainability of LDA carrier and bacteriostatic effects, and their clinical, radiographical, and immunohistochemical efficacy on experimentally induced peri-implantitis model. Macroscopic inspection of residual drug agents showed significantly prolonged retention of CA microspheres (MC and MP) compared to PG microspheres. Concomitantly, a prolonged bacteriostatic effect was observed from MC compared to PG. All groups presented significantly reduced mean PPD at T3 compared to T1 and T2 while MC was the only group that showed a significant reduction of mean PPD between each measurement period. PG presented significantly improved GI and BOP (%) at T2 and T3 compared to baseline. The periapical radiographs showed a negligible marginal bone level change in all groups during the treatment period (−0.06 mm (MP) to −0.25 mm (PG)). IHC cell marker analysis showed comparable results between the groups.

The two different constituents of minocycline-contained microspheres (CA and PG) exhibited a distinct degradability rate in the peri-implantitis induced model. In the CA microsphere, negatively charged alginate polysaccharide is surrounded by positively charged chitosan polysaccharide [31]. The two polysaccharides are bonded by ionic coacervation with the aid of calcium-chloride cross-linking agent [19]. The degradation rate of the CA microsphere can be controlled by the amount of chitosan concentration in the microsphere [19] owing to the fact that chitosan is physiologically degraded by lysozyme and bacterial enzymes in the colon [32]. On the other hand, the PG microsphere, composed of polymethacrylate, triacetin, and hydroxyethyl cellulose, is hydrophilic and quickly dissolves after administration. Therefore, contained minocycline is immediately released and the surrounding minocycline concentration is instantly increased then decreased in a reciprocal-like manner [18].

The drug sustainability evaluation of this study showed comparable results with the previous mucositis-induced study [20] in both carrier and bacteriostatic effect sustainability. Macroscopic inspection of the two studies revealed that CA-microsphere LDA remain longer in the gingival sulcus compared to PG-microsphere LDA. Furthermore, prolonged sustainability of LDA had led to elongated bacteriostatic durability in both studies.

Progression of peri-implantitis can be measured by increased probing depth due to gingival swelling or peri-implant bone loss [1]. Clinical assessment of this study showed significantly reduced intragroup mean PPD at T3 compared to baseline in all of the treatment groups (*p* < 0.05). This implies that the disease has not been progressed during the treatment period. Among the treatment groups, MC was the only group that showed a significantly reduced PPD after each delivery (*p* < 0.05) while PG was the only group that showed significantly reduced BOP (%) and GI scores at T2 and T3 compared to baseline. However, despite these intragroup changes, all clinical outcomes were comparable between the groups. 

Progression of marginal bone loss is a distinguishable clinical characteristic of peri-implantitis from mucositis [1]. In the radiographical analysis, all treatment groups showed minimal marginal bone level changes during the treatment period ranging from −0.07 (MC) to −0.25 mm (PG). Taking into account that the mucositis study showed mean marginal bone loss occurred at a range of −0.25 to −0.44 mm, minimal bone level changes that occurred in this study indicate that all treatment groups have impeded the disease progression.

The radiographical outcome and PPD measurements in this study showed that mechanical debridement with or without LDA may impede the disease progression. However, BOP (%) distribution is still considerably high at T3 (44.5 to 77.8%), and that higher BOP (%) distribution is observed from CA microspheres (MC and MP) when compared to PG or Control (Table 1). A previous in vitro study asserted on the possible negative impingement of CA microspheres in the healing process after treatment due to its long sustainability remained in the gingival sulcus [33]. The authors carefully speculate that prolonged retention of the carrier may influence the soft tissue healing process.

Among the three monoclonal antibodies, IgG, an antibody of B-cells and plasma cells, represents chronic inflammation severity. Although IHC cell marker analysis showed comparable results across the treatment groups, MC showed a lower tendency of distribution among other treatment groups which coincides with the mucositis study. A closer examination of IHC stained slides revealed that most of the inflammatory cells were clustered at the junction of abutment and fixture. Infiltrated connective tissue (ICT) was mostly confined within 0.5 mm horizontally from the contact of abutment and fixture. Inflammatory cells including plasma and multinucleated cells were occasionally detected in the connecting tissue near the bone crest. Furthermore, the thick ulcerated pocket epithelium was visible at the coronal portion of the most inflamed part of ICT. Once it has passed, the thin lining of an epithelial barrier was stretched to the coronal part of the bone crest. Although these histomorphologic findings have been discussed in a previous experimentally induced peri-implantitis using external connection design implants [26,34], the present experiment revealed that despite the platform switching, the anatomical ‘step’ created between abutment and fixture provides a haven for microbial penetration.

A recent randomized clinical trial conducted in patients with peri-implantitis reported that the repeated administration of LDA during and after the surgical treatments has a positive effect on the clinical and radiographical outcomes [17]. This animal study had also repeatedly administered LDA at a 1-month interval. In the clinical evaluations, all the treatment groups presented significantly reduced PPD (*p* < 0.05) during the second interval (T2–T3) compared to the first (Baseline–T2) (Table 1). In addition, the PG group continued to show a reduction in BOP (%) and GI scores from Baseline to T3. Improved clinical outcomes were much more evident when compared to the mucositis study where there were no intragroup differences in clinical outcomes over the treatment period. 

The authors hypothesized that prolonged sustainability of LDA could promote a longer bacteriostatic effect and would eventually improve treatment outcomes. Although improved intragroup clinical outcomes were observed over the treatment period, the treatment efficacies were comparable between the groups. The treatment outcome may have been influenced by some of the limitations of this study. Firstly, the total amount of minocycline contained in each 0.5 g syringe (10 mg of minocycline hydrochloride) in MC and PG are equivalent. Therefore, despite the prolonged bacteriostatic durability of MC, the cumulative effect of the two types of LDA may be equivalent. For this reason, a future study with higher minocycline concentration with CA microsphere is recommended. Secondly, oral hygiene control was only maintained on the supragingival part of the healing abutments with a hydrogen peroxide-soaked gauze during the observation period to not interfere with the sulcular environment and to accurately evaluate the drug sustainability. Therefore, cautious interpretation of the remaining BOP is recommended.

This in vivo study evaluated carrier sustainability of the two different constituents of microspheres and longevity of bacteriostatic effects with respect to their degradation property and their clinical, radiographical, and immunohistochemical efficacies. Following the ARRIVE guidelines, only short-term clinical and radiographical outcomes were evaluated. Further studies are recommended to evaluate long-term treatment outcomes.

## 5. Conclusions

Within the limitations of this study, prolonged retention of CA microspheres was observed, and the longer bacteriostatic effect was observed from the MC group. Mechanical debridement with adjunct LDA therapy may impede peri-implantitis progression, however, prolonged drug action did not lead to improved treatment outcome. 

## Figures and Tables

**Table 1:** Clinical data (± S.D.) at different time points.

Clinical Parameter	Group	Baseline (T1)	4 Weeks (T2)	8 Weeks (T3)
Mean PPD (mm)	MC	3.81 ± 0.99	3.06 ± 0.59 ^‡^	2.00 ± 0.62 ^‡^,*
	MP	3.81 ± 0.91	3.67 ± 0.83	2.17 ± 0.58 ^‡^,*
	PG	3.44 ± 0.30	3.19 ± 0.35	2.42 ± 0.60 ^‡^,*
	Control	3.47 ± 0.41	3.11 ± 0.62	2.17 ± 0.65 ^‡^,*
*p*-value		0.980	0.506	0.782
Mean GI	MC	1.92 ± 0.13	1.78 ± 0.12	1.78 ± 0.18
	MP	1.92 ± 0.13	1.72 ± 0.16	1.53 ± 0.35
	PG	1.89 ± 0.12	1.69 ± 0.18 ^‡^	1.44 ± 0.34 ^‡^
	Control	1.81 ± 0.22	1.58 ± 0.08	1.61 ± 0.31
*p*-value		0.720	0.154	0.491
Mean BOP (%)	MC	91.7 ± 12.7	77.8 ± 12.2	77.8 ± 18.3
	MP	91.7 ± 12.7	69.7 ± 14.9	69.5 ± 29.4
	PG	88.8 ± 12.4	69.5 ± 17.7 ^‡^	44.5 ± 34.1 ^‡^
	Control	91.5 ± 8.5	64.0 ± 11.4 ^‡^	50.2 ± 33.3 ^‡^
*p*-value		0.955	0.433	0.414
Mean PLI	MC	2.70 ± 0.34	2.33 ± 0.62	1.45 ± 0.29 ^‡^,*
	MP	2.75 ± 0.25	2.39 ± 0.57	1.31 ±0.35 ^‡^,*
	PG	2.75 ± 0.25	2.50 ± 0.53	1.25 ± 0.48 ^‡^,*
	Control	2.67 ± 0.26	1.91 ± 0.98	1.17 ± 0.37 ^‡^
*p*-value		0.890	0.383	0.585

^‡^—significantly different from the baseline within each group (*p* < 0.05). *—significantly different from T2 within each group (*p* < 0.05). *p*-value—clinical parameters compared (Kruskal–Wallis test) between the groups at each time point.

**Table 2:** IHC cell marker analysis: high positive + positive score combined ROI 0.5 × 0.5 mm (units in %).

		MC	MP	PG	Control	*p*-Value
CD 3 (%)	A/F—Top	7.14 ± 2.84	9.24 ± 6.09	9.49 ± 6.84	5.99 ± 3.65	0.516
	A/F—Bottom	8.20 ± 3.78	9.34 ± 5.89	7.69 ± 6.28	6.73 ± 4.34	0.824
	Total mean	7.67 ± 3.39	9.29 ± 6.00	8.59 ± 6.63	6.34 ± 4.01	
CD 20 (%)	A/F—Top	9.21 ± 4.67	8.28 ± 4.58	8.76 ± 9.07	9.93 ± 4.67	0.388
	A/F—Bottom	8.02 ± 4.14	7.48 ± 4.50	8.06 ± 4.15	7.17 ± 5.32	0.887
	Total mean	8.48 ± 4.39	7.85 ± 4.56	8.43 ± 7.19	8.55 ± 5.19	
Ig G (%)	A/F—Top	8.41 ± 4.99	9.75 ± 6.29	11.72 ± 10.99	13.10 ± 9.90	0.898
	A/F—Bottom	4.97 ± 3.25	7.93 ± 8.23	8.45 ± 5.26	9.57 ± 8.80	0.467
	Total mean	6.59 ± 4.50	8.84 ± 7.38	10.00 ± 8.63	11.33 ± 9.53	

*p*-value—IHC cell marker distribution compared (Kruskal–Wallis test) between the groups at top and bottom ROI.

## Appendix A

**Table A1:** Panels of monoclonal antibodies used for IHC staining.

Antibody	Specificity	Dilutions	Source
CD3	T cells	1:200	Abcam
CD20	B cells	1:800	Thermo-Fisher scientific
IgG	Plasma/B Cells	1:800	Cloud-Clone corp.

**Table A2:** Mean radiographic interval changes (mm) at different time points.

	ΔSurgery − T1	ΔT1 − T2	ΔT2 − T3	ΔT1 − T3
MC	−1.39 ± 0.69	0.17 ± 0.28	−0.24 ± 0.25	−0.07 ± 0.23
MP	−1.61 ± 1.03	0.12 ± 0.29	−0.06 ± 0.36	−0.06 ± 0.53
PG	−0.86 ± 0.24	−0.15 ± 0.24	−0.09 ± 0.46	−0.25 ± 0.42
Control	−1.09 ± 0.60	0.03 ± 0.18	−0.16 ± 0.44	−0.13 ± 0.56
*p*-value	0.445	0.357	0.880	0.843

*p*-value—marginal bone level changes compared (Kruskal–Wallis test) between the groups at each interval.
